# Structural characterization of S100A15 reveals a novel zinc coordination site among S100 proteins and altered surface chemistry with functional implications for receptor binding

**DOI:** 10.1186/1472-6807-12-16

**Published:** 2012-07-02

**Authors:** Jill I Murray, Michelle L Tonkin, Amanda L Whiting, Fangni Peng, Benjamin Farnell, Jay T Cullen, Fraser Hof, Martin J Boulanger

**Affiliations:** 1 Department of Chemistry, University of Victoria, PO Box 3065, STN CSC, Victoria, BC, V8W 3P6, Canada; 2 Department of Biochemistry and Microbiology, University of Victoria, PO Box 3055, STN CSC, Victoria, BC, V8W 3P6, Canada; 3 School of Earth and Ocean Sciences, University of Victoria, PO Box 3065, STN CSC, Victoria, BC, V8W 3P6, Canada

**Keywords:** S100A15, S100A7, Zinc-binding, EF hand, X-ray crystallography

## Abstract

**Background:**

S100 proteins are a family of small, EF-hand containing calcium-binding signaling proteins that are implicated in many cancers. While the majority of human S100 proteins share 25-65% sequence similarity, S100A7 and its recently identified paralog, S100A15, display 93% sequence identity. Intriguingly, however, S100A7 and S100A15 serve distinct roles in inflammatory skin disease; S100A7 signals through the receptor for advanced glycation products (RAGE) in a zinc-dependent manner, while S100A15 signals through a yet unidentified G-protein coupled receptor in a zinc-independent manner. Of the seven divergent residues that differentiate S100A7 and S100A15, four cluster in a zinc-binding region and the remaining three localize to a predicted receptor-binding surface.

**Results:**

To investigate the structural and functional consequences of these divergent clusters, we report the X-ray crystal structures of S100A15 and S100A7D24G, a hybrid variant where the zinc ligand Asp24 of S100A7 has been substituted with the glycine of S100A15, to 1.7 Å and 1.6 Å resolution, respectively. Remarkably, despite replacement of the Asp ligand, zinc binding is retained at the S100A15 dimer interface with distorted tetrahedral geometry and a chloride ion serving as an exogenous fourth ligand. Zinc binding was confirmed using anomalous difference maps and solution binding studies that revealed similar affinities of zinc for S100A15 and S100A7. Additionally, the predicted receptor-binding surface on S100A7 is substantially more basic in S100A15 without incurring structural rearrangement.

**Conclusions:**

Here we demonstrate that S100A15 retains the ability to coordinate zinc through incorporation of an exogenous ligand resulting in a unique zinc-binding site among S100 proteins. The altered surface chemistry between S100A7 and S100A15 that localizes to the predicted receptor binding site is likely responsible for the differential recognition of distinct protein targets. Collectively, these data provide novel insight into the structural and functional consequences of the divergent surfaces between S100A7 and S100A15 that may be exploited for targeted therapies.

## Background

The S100 calcium-binding protein family of vertebrate, metal-regulated proteins plays pivotal roles in a wide variety of intracellular and extracellular functions including cell growth, inflammation, membrane remodeling and chemotaxis [1,2] and are implicated in many diseases including cancer [3-6]. Hallmarks of the S100 protein family include their small size (~ 100 aa) and the presence of a canonical (calmodulin-like) EF-hand motif and a non-canonical (S100-specific) EF-hand motif [7]. Additional complexity in S100 proteins is derived from their ability to adopt a non-covalent anti-parallel homo/heterodimers that can further associate to yield higher order multimers such as tetramers and hexamers [3,5,8,9].

While most S100 proteins share moderate sequence identity, S100A7 and S100A15 are 93% identical in sequence yet intriguingly display divergent functions. S100A7, for example, is implicated in inflammatory skin diseases and in several cancers including breast cancer [4,10-13] where it is associated with aggressive estrogen receptor negative tumors and poor prognosis [14,15]. Intracellular S100A7 has also been shown to interact with the multifunctional signaling protein Jab1, resulting in translocation of Jab1 to the nucleus where it mediates S100A7 tumorigenic effects [16,17]. While S100A15 is also highly expressed in psoriatic lesions [18,19], it displays distinct localizations in skin and breast, divergent functions in epithelial maturation and skin inflammation [19,20] and it is currently unknown whether S100A15 contributes to breast cancer progression [21]. S100A7 and S100A15 are also capable of binding different surface receptors [5]. S100A7 interacts with RAGE (receptor for advanced glycated end products) to mediate chemotaxis of leukocytes [20,22] while S100A15 appears to mediate chemotaxis through an unidentified G protein coupled receptor [20].

The ability of many S100 proteins to coordinate zinc is central to their function [23]. For example, the ability of S100A7 to engage RAGE and exert antimicrobial effects are both dependent on zinc [20][24]. These observations suggest a regulatory role although the zinc coordination geometry and affinity suggest a structural role [25]. It is not yet known whether zinc binding plays a structural or regulatory role in S100A7 and S100A15 function. To our knowledge, there is no data to suggest S100A15 function is zinc-dependent. Thus far, two types of S100-family zinc sites have been proposed [23]. In the first type (‘His-Zn’), the zinc is coordinated by 3 histidines and an aspartate (*i.e.* S100A7 and S100A12) or with four histidines (*i.e.* S100B). The second type (‘Cys-Zn’) is proposed to contain primarily cysteines as the zinc-coordinating residues though no structure of an S100 ‘Cys-Zn’ binding site has been reported. Ultimately defining the zinc binding capacity for S100A15 is crucial to understanding its function and appropriately categorizing it within the S100 family of proteins. To this end, we report the structural characterization of S100A15 and an Asp24Gly variant of S100A7 to 1.7 Å and 1.6 Å, respectively. These data reveal an unexpected structural compensation to retain zinc binding in S100A15 and a structural rationale for the inability of S100A15 to coordinate RAGE and the prediction that S100A15 will not bind Jab1.

## Methods

### Protein production and purification

S100A15 (S100A7 template with the following substitutions - D24G, R21G, D27E, N51H, T52I, D56T, T83A) and S100A7 D24G were generated using using Quikchange mutagenesis (Stratagene) of wildtype S100A7 [10] and recombinantly produced as a thioredoxin fusion with an N-terminal hexa histidine tag. Protein production was performed in *E. coli* Rosetta-Gammi B in Overnight Express™ Instant TB Medium (Novagen) overnight at 37°C. The cells were harvested, resuspended in binding buffer (20 mM HEPES pH 8, 30 mM imidazole, 1 M NaCl, and 50 μM zinc chloride), lysed using a French press, applied to a His-trap column and eluted using an increasing gradient of imidazole to 500 mM. Fractions were analyzed with SDS-PAGE, pooled based on purity, concentrated and the histidine tag removed by thrombin digestion prior to a final purification step using superdex Sx75 gel filtration column. Due to the absence of Tryptophan or phenylalanine residues, protein concentration was calculated based on amino acid analysis.

### Crystallization, data collection, processing and structure solution

Crystallization was performed at 18 °C using the sitting drop vapour diffusion method. S100A15 was crystallized at 26 mg/ml in 45–50 mM zinc acetate dihydrate, 18-20% PEG 3350 and S100A7D24G was crystallized at 19 mg/ml in 0.2 M ammonium sulfate, 0.1 M BisTris pH 5.5, 20-25% PEG 3350. Diffraction data were collected on beamline 9–2 at SSRL and processed with d*trek [26]. Data collection and refinement statistics are presented in Table 1. Refinement was performed with the CCP4 suite of programs [27]. The initial phases were obtained by molecular replacement (MR) using MOLREP [28] with the monomeric form of native S100A7. Solvent atoms were selected using COOT [29] and the structure refined with REFMAC [28]. Stereo-chemical analysis of the refined structures was performed with PROCHECK and SFCHECK in CCP4 [27]. Overall 5% of the reflections were set aside for calculation of R_free_.

**Table 1:** Data collection and refinement statistics

**A. Data collection**	**S100A15**	**S100A7D24G**
Spacegroup	I2	P4_3_2_1_2
a, b, c (Å)	52.03, 33.47, 64.05	51.48, 51.48, 117.23
α, β, γ (deg.)	90, 90.81, 90	90, 90, 90
Wavelength	0.9794	0.9794
Resolution range (Å)	40.66-1.70 (1.79-1.70)	38.68-1.60 (1.69-1.60)
Measured reflections	44302	203068
Unique reflections	12285	21100
Redundancy	3.6 (3.6)	9.6 (9.8)
Completeness (%)	99.6 (100.0)	99.6 (98.4)
*I/σ(I)*	8.7 (2.5)	15.3 (2.6)
R_merge_^a^	0.071 (0.385)	0.048 (0.430)
**B. Refinement**		
Resolution (Å)	28.15-1.70 (1.74-1.70)	34.76-1.60 (1.64-1.60)
R_cryst_^b^	0.199 (0.313)	0.190 (0.250)
R_free_^c^	0.230 (0.328)	0.225 (0.274)
No. of atoms		
Protein	767	773
Solvent	110	148
Calcium	1	1
Zinc	2	1
Chloride	1	1
B-values (Å^2^)		
Protein	30.29	23.42
Solvent	45.84	41.57
Calcium	23.60	19.85
Zinc	30.52	20.80
Chloride	35.94	24.99
r.m.s.d		
Bond lengths (Å)	0.013	0.016
Bond angles (deg.)	1.321	1.533
Ramachandran statistics		
Most favoured	97.87	98.91
Allowed	2.13	1.09
Disallowed	0	0

### Protein data bank accession number

Coordinates and structure factor accession codes are as follows: S100A15 (4AQI, r4aqisf) and S100A7D24G (4AQJ, r4aqjsf).

## Results and discussion

### S100A15 retains the ability to coordinate zinc

**Figure 1:**
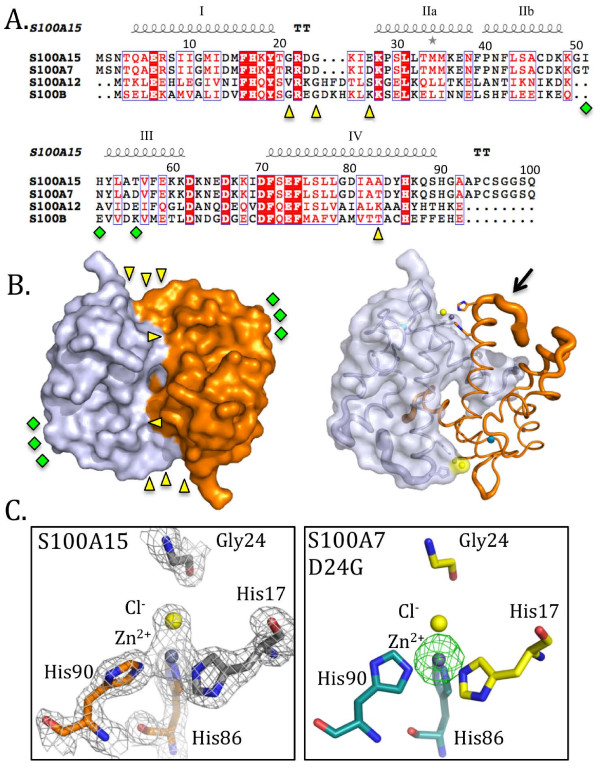
**S100A15 binds zinc with distorted tetrahedral geometry.A.** Sequence alignment of S100A15 (NP_789793.1), S100A7 (CAG46961.1), S100A12 (NP_005612.1) and S100B (CAG46920.1). Divergent residues between S100A15 and S100A7 are denoted as yellow triangles (zinc binding cluster) and green diamonds (predicted receptor binding cluster). **B.** Surface representation of S100A15 with one monomer shown in sky blue and the other in orange. Black arrow in the right panel denotes the flexibility of the surface loop to which the His90 zinc ligand is attached. **C.** Left panel – Fo-Fc electron density of the zinc binding site of S100A15 calculated at 1.5 sigma with zinc shown as a grey sphere and the chloride ligand shown as a yellow sphere. Right panel – Anomalous difference electron density map of S100A7D24G using data collected at the zinc edge of 1.2482 nm calculated at 5 sigma confirming that the bound metal is zinc. All figures were generated with PyMol.

To investigate the differences in zinc binding between S100A7 and S100A15, we recombinantly produced human S100A15 in *E. coli* and purified the protein to homogeneity using nickel affinity chromatography. Similar to S100A7, S100A15 eluted as a stable dimer from a size exclusion column (SEC) indicating that the seven divergent residues (*R*21G*D*24G*D*27E*T*83A*T*51I*N*52H and *D*56T – S100A7 residues are shown in italics and S100A15 residues are underlined) (Figure 1A) did not disrupt the multimeric structure. S100A15 crystallized as a monomer in the asymmetric unit of the monoclinic cell with the dimer generated by crystallographic symmetry (Figure 1B). Molecular replacement was used to solved the S100A15 structure with native S100A7 [9] as the search model and all waters, zinc and calcium ions removed and the 7 divergent residues (highlighted as yellow triangles and green diamonds in Figure 1A and B) mutated to alanine. The S100A15 structure was refined to a resolution of 1.7 Å and displayed an overall root mean squared deviation (r.m.s.d) of 0.661 Å^2^ over 96 Cα atoms relative to S100A7.

During the initial refinement stages of the S100A15 structure an 8 sigma difference electron density peak was observed that overlaid with the bound zinc in S100A7 (Figure 1C). Accordingly, a zinc ion was modeled and refined at full occupancy resulting in a B-factor of 30.52 Å^2^. Thus, it was apparent that substitution of Gly24 in S100A15 for the zinc ligand Asp24 (S100A7) did not abrogate zinc binding. Remarkably, the coordination geometry of the three histidine ligands (His86 and His90 - monomer 2) and His17 - monomer 1) (Figure 1C) is virtually unchanged between S100A7 (109°±9°) and S100A15 (109°±3°). Of these ligands, only His90 displayed a measurable shift with a 15° reorientation and a shift of the imidazole ring NE2 and ND1 by 1.1 Å and 2.2 Å, respectively, likely due to the position of His90 on a mobile surface loop (Figure 1B – black arrow). To complete the distorted tetrahedral geometry of the bound zinc in S100A15, a chloride ion was modeled, which refined to a distance of 2.1 Å from the zinc and a B factor of 25.4 Å^2^. While water molecules are commonly observed as labile ligands in zinc coordination sites, we elected to model a chloride ion using the B factor as a guide and based on the fact that chloride was the dominant anion in our buffer. While Asp24 has been previously observed as a bindentate ligand [30], we measured Zn-O bond distance of 2.04 Å (OD1) and 2.69 Å (OD2). The shorter bond length between Zn and Asp24 OD1 suggests that S100A7 Asp 24 likely acts as a monodentate ligand, and that both S100A15 and S100A7 bind zinc in a 4 coordinate geometry. In support of the relevance of the observed zinc coordination by S100A15, we obtained preliminary isothermal titration calorimetry data by 1) treating with Chelex resin to remove zinc and 2) back-titrating with ZnCl_2_, to allow direct measurement of binding. These preliminary results show nearly identical dH values of −12.3 ± 0.1 kcal/mol and −12.1 ± 0.1 kcal/mol for S100A7 and S100A15, respectively, suggesting similar strengths of Zn coordination by both proteins in spite of the change of coordination environment. Zn affinities determined by ITC (~10^9^ M^–1^ for S100A7, and 4 x 10^8^ M^–1^ for S100A15) vary depending on buffer composition, but are generally within five-fold of each other under a given set of conditions. The construction of pseudopolarograms on S100A7 and S100A15 samples using anodic stripping voltammetry, and the analysis of resulting half-wave potential shifts also indicate that both proteins bind zinc with similar affinity [31]. Full solution-phase data will be reported in due course.

### Replacement of Asp24 (S100A7) with a glycine (S100A15) is sufficient to produce a zinc-binding site unique among S100 proteins

To confirm that the substitution of Asp24 with a glycine was solely responsible for the unique zinc-binding site observed in S100A15, we generated the D24G variant of S100A7. S100A7D24G eluted as a dimer from the SEC, and crystallized with one monomer in the asymmetric unit of the tetragonal cell. Diffraction data was collected to a resolution of 1.6 Å (Table 1) and the structure solved by molecular replacement. The S100A7D24G and S100A15 structures aligned closely with an r.m.s.d of 0.719 Å over 96 Cα atoms. Subtle adjustments were observed in the orientation of the histidine ligands, due to differential packing of surface loops between the monoclinic and tetragonal crystal forms. To validate the modeling of zinc at the dimer interface, we calculated an anomalous difference map using data collected at the zinc edge of 1.2482 nm. A large anomalous difference peak (>12 σ) was observed where the zinc had been modeled confirming the identity of the bound metal as zinc (Figure 1C – right panel). While crystallization of S100A15 was completed in the presence of zinc, S100A7D24G was crystallized in the absence of zinc, and yet zinc binding was observed in the same geometry. These observations indicate that the zinc-binding observed in the S100A15 is not an artifact. Similar to S100A15, a chloride ion was modeled as the exogenous fourth ligand to complete the distorted tetrahedral geometry. The incorporation of a dissociable ligand such as chloride may enable a binding partner to donate a fourth ligand thereby ensuring a tighter ligand-receptor complex. A second zinc atom was observed in the S100A15 structure (at Glu36), which was not observed in the S100A7D24G mutant. We expect this is an artifact as S100A15 WT was crystallized in high concentrations (50 mM) of zinc while S100A7D24G was crystallized in the absence of zinc.

**Figure 2:**
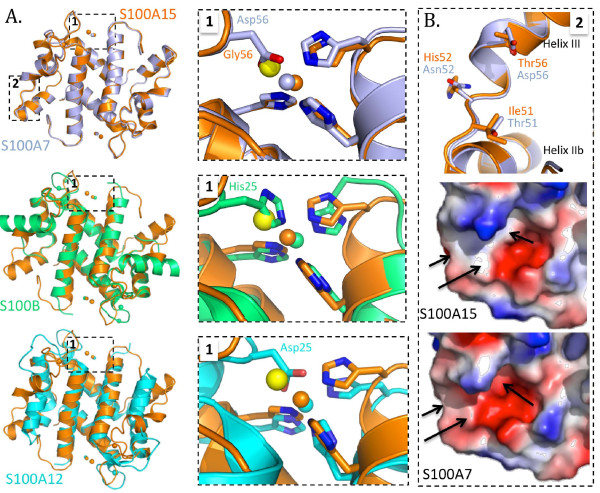
**Zinc binding in S100 proteins.A.** Comparison of zinc binding sites between S100A15 and S100A7 (upper panel), S100B (middle panel) and S100A12 (lower panel). Note the unique strategy employed in S100A15 to incorporate an exogenous ligand to complete the distorted coordination geometry of the bound zinc. **B.** Structural divergence between S100A15 and S100A7 localized to the predicted receptor-binding surface highlighted by the black arrows representing the three divergent residues. Note the decreased acidity in S100A15.

Analysis of zinc binding sites between S100A15, S100A7, S100B and S100A12 reveal highly conserved coordination geometry for the metal positioned at the dimer interface. While sequence analysis shows that S100A12 and S100B encode a glycine at the equivalent sequence (Figure 1A) position to 24 in S100A15, a three-residue insertion (relative to S100A15 and A7) results in a histidine (S100B) or an aspartate (S100A12) being correctly positioned to serve as the fourth zinc ligand (Figure 2). Thus, it appears that multiple different strategies are used by S100 proteins to maintain the distorted coordination geometry of the zinc bound at the dimer interface consistent with a central functional role. In fact, the strict conservation of the zinc site is remarkable especially in the context of structural divergences relative to S100A15 (r.m.s.d for S100B of 0.802 Å over 118 Cα atoms, and S100A12 of 2.109 Å over 113 Cα atoms) with substantial reorganization in nearby surface loops (Figure 2A). It is not clear why the zinc binding affinities of rat S100B (0.094 μM) and human S100A12 (4.5 μM) are much higher than human S100A7 (100 μM) [32-34].

### Altered surface chemistry on S100A15 may explain the lack of RAGE and predicted Jab1 binding

The second cluster of amino acid differences (*T*51I*N*52H and *D*56T - S100A7 residues are shown in italics and S100A15 residues are underlined) between S100A7 and S100A15 is localized to an ordered loop between helices IIb and III and extending into the N-terminal portion of helix III. The center of this cluster is positioned approximately 24 Å from the closest zinc-binding site. Intriguingly, this region in S100A7 has been implicated in mediating interaction with Jab1 [8,9,17] and between S100B and RAGE [35]. The inability of S100A15 to bind RAGE [20], and the prediction that S100A15 lacks a functional Jab1 binding site [8,36], suggests that this localized cluster of residues is a hot spot surface for ligand-receptor interactions in the S100 family of proteins. Functional consequences based on altered secondary structure have been observed in S100A7 where mutation of Asp56 to Gly resulted in shortening of helix III [8]. However, no such changes were observed in S100A15 and overlay of helix III (amino acids 50–62) of S100A7 and S100A15 yielded an r.m.s.d of only 0.219 Å over 9 Cα atoms. Overall, the replacement of Asp56 with Thr and Asn52 with His substantially alters the surface chemistry and may explain the inability of S100A15 to bind RAGE.

Despite being capable of specific interactions with unique targets, the emerging data on S100-RAGE interactions suggest that S100 proteins have evolved to also interact with a common receptor, RAGE, *via* unique structural binding sites and in distinct multimeric states [35,37-39]. Adding to the combinatorial possibilities of S100-RAGE interactions is the observation that the RAGE is capable of multimerization: S100B tetramers interact with RAGE dimers, S100B dimers interact with RAGE monomers [40], and S100A12 hexamers interact with RAGE tetramers [37,38]. However, close examination of the amino acid sequences and structures of S100B, S100A12, S100A7 and S100A15 (Figures 1A and 2B) offers little to explain why S100A15 does not bind RAGE while other S100 proteins do. Extensive scanning mutagenesis or structural characterization of the complexes will ultimately be required to define the core features that promote macromolecular assembly.

## Conclusions

Zinc-coordination is a dynamic process and can play a role in the regulation of protein function [25]. Recent studies have suggested that misregulation of zinc homeostasis is associated with cancer [41]. Distinct metal binding and protein-interacting functions have been described for S100A7 and S100A15 despite their 93% sequence identity. Zinc is required for several S100A7 functions, including antimicrobial action and RAGE binding. S100A15 does not bind RAGE and a zinc-dependent function has not yet been identified, which has led to the suggestion that S100A15 does not bind zinc. Here we demonstrate that S100A15 does in fact coordinate zinc in a distorted tetrahedral geometry with an exogenous ligand that is unique to S100 proteins. These data place S100A15 as the first member of what is likely to be a growing subgroup of S100 proteins. Further studies are required to establish the mechanism of receptor interactions with the S100 proteins, but divergent solutions to maintain zinc binding is undoubtedly going to play a central role.

## Competing interests

The authors declare that they have no competing interests.

## Author’s contributions

JM, FH and MB conceived of the study and along with MT, AW and JC designed the experiments. JM and MB performed the crystallization, data collection and structure solution. JM, MT and BF produced the recombinant S100 proteins. AW and JC performed the solution binding studies. JM and MB wrote the manuscript with editing contributed by MT, FP and FH. All authors read and approved the final manuscript.
